# Ovarian endometrioid adenofibroma in a perimenopausal woman with intermittent vaginal bleeding: a case report

**DOI:** 10.3389/frph.2026.1839813

**Published:** 2026-09-28

**Authors:** Zhigang Niu, Hongwei Liang, Ning Wang, Jiazhen Huang

**Affiliations:** 1 Department of Obstetrics and Gynecology, The Second Affiliated Hospital of Dalian Medical University, Dalian, China; 2 Department of Radiotherapy Oncology, The Second Affiliated Hospital of Dalian Medical University, Dalian, China

**Keywords:** case report, diagnosis, endometrioid adenofibroma, ovarian neoplasms, therapy

## Abstract

Ovarian endometrioid adenofibroma (EA) is an exceptionally rare benign epithelial tumor of the ovary with nonspecific clinical and imaging manifestations, and its diagnosis relies mainly on pathological examination. This case describes a 55-year-old perimenopausal woman with a 1-year history of intermittent vaginal bleeding and a progressively enlarging right adnexal mass. Imaging revealed a solid mass in the right adnexal region with minimal blood flow signal. Serum tumor markers were within normal ranges, and the sex hormone profile showed no evidence of estrogen excess. Serial ultrasonography showed no interval increase in endometrial thickness; therefore, a causal relationship between the ovarian tumor and the abnormal bleeding could not be established. The patient underwent laparoscopic total hysterectomy and bilateral salpingo-oophorectomy. Postoperative uterine pathology demonstrated proliferative endometrium and endometrial polyps, providing a plausible uterine explanation for the bleeding. Histopathological examination of the ovarian lesion confirmed benign endometrioid adenofibroma with focal epithelial crowding but without cytological atypia, increased mitotic activity, or destructive stromal invasion. The postoperative course was uneventful, and no abnormal findings were detected during the 11-month follow-up. EA lacks specific clinical manifestations, and definitive diagnosis depends on histopathological and immunohistochemical evaluation. Management is primarily surgical, but the extent of surgery should be individualized according to patient age, menopausal status, fertility considerations, and preoperative suspicion of malignancy. Although the prognosis of benign EA is generally favorable, careful follow-up may be considered in cases with atypical histological features. This case highlights the diagnostic challenge of ovarian EA and emphasizes its consideration in the differential diagnosis of solid adnexal masses in peri- and postmenopausal women, particularly when imaging findings raise concern for malignancy.

## Introduction

Ovarian endometrioid adenofibroma (EA) is an exceptionally rare benign epithelial tumor characterized by Müllerian/endometrioid epithelial differentiation, accounting for only approximately 1% of ovarian epithelial tumors (1–3). Due to its rarity and nonspecific clinical manifestations, imaging findings, and tumor marker profiles, EA is often difficult to diagnose preoperatively and is frequently identified only after pathological examination (1, 2). Although typical benign EA generally has a favorable prognosis, rare atypical, proliferative, borderline, or malignant counterparts have been described. These uncommon variants may exhibit increased epithelial proliferation or overlapping histopathological features with endometrioid carcinoma; however, typical benign EA should not be regarded as a premalignant lesion. Rare associations between EA and mesonephric-like adenocarcinoma have also been reported, although their biological relationship remains unclear (4, 5).

Herein, we report a case of ovarian endometrioid adenofibroma in a perimenopausal woman with intermittent vaginal bleeding. Through discussion of the clinical presentation, pathological characteristics, diagnostic considerations, and management of this rare tumor based on the present case and relevant literature, we aim to improve recognition of EA and enhance diagnostic accuracy in patients presenting with solid adnexal masses.

## Case presentation

A 55-year-old perimenopausal woman was admitted to our hospital on January 3, 2025, because of intermittent vaginal bleeding for nearly 1 year. Gynecological ultrasonography performed at an outside hospital on July 9, 2024 showed an endometrial thickness of approximately 6 mm and a right adnexal hypoechoic mass measuring approximately 56 mm × 54 mm; the right ovary was not clearly identified. Repeat ultrasonography on December 30, 2024 demonstrated enlargement of the right adnexal mass to approximately 71 mm × 65 mm × 60 mm, with newly noted multiple coarse hyperechoic foci and a small amount of blood flow signal on color Doppler imaging. The endometrial thickness remained approximately 6 mm without interval thickening. Representative preoperative ultrasonographic images obtained on December 30, 2024 are provided in Supplementary Figure S1. Gynecological examination revealed a normal vulva, unobstructed vagina, smooth cervix, and negative cervical motion tenderness. On bimanual examination, a mass approximately 6 cm in diameter was palpable on the right side of the uterus, with limited mobility and a tough consistency. No obvious abnormalities were detected in the left adnexal region. Although the lesion was described as hypoechoic on ultrasonography, the contemporaneous ultrasound report interpreted it as a solid right adnexal mass rather than a cystic lesion. Subsequent MRI likewise demonstrated a predominantly solid mass. Pelvic magnetic resonance imaging (MRI) without contrast (1.5 T) (Figure 1) demonstrated a normal uterus and a large right adnexal mass measuring approximately 65 mm × 55 mm × 62 mm. The detailed imaging characteristics are presented in Figure 1. Laboratory investigations showed normal serum tumor marker levels, including CA125 (5.77 U/mL; reference range <35.0 U/mL) and HE4 (54.99 pmol/L; reference range for postmenopausal women <140.0 pmol/L). The sex hormone profile showed luteinizing hormone (LH) 9.21 mIU/mL (reference range: 10–80 mIU/mL), follicle-stimulating hormone (FSH) 26.33 mIU/mL (reference range: 25–135 mIU/mL), prolactin (PRL) 89.31 *μ*IU/mL (reference range: 60–620 *μ*IU/mL), testosterone (T) 0.16 ng/mL (reference range: 0.08–0.60 ng/mL), estradiol (E2) 18.30 ng/L (reference range: <30 ng/L), and progesterone (P) 0.75 ng/mL (reference range: <1.0 ng/mL). The patient denied a history of hypertension, diabetes mellitus, coronary heart disease, or malignant tumors.

**Figure 1:**
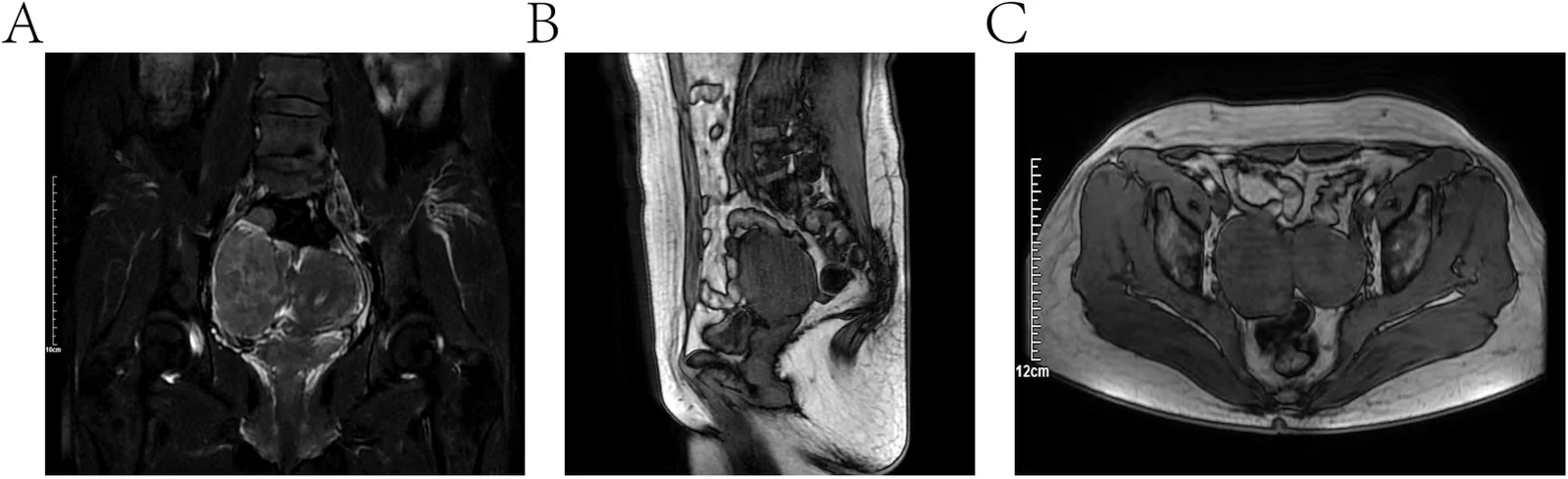
Non-contrast pelvic magnetic resonance imaging (MRI) findings of the patient obtained using a 1.5-T scanner. **(A)** Coronal pelvic MRI showing a well-defined round soft-tissue mass in the right adnexal region, with normal uterine morphology and unremarkable left adnexa. **(B)** Sagittal pelvic MRI demonstrating the right adnexal mass, with preserved uterine morphology and no definite evidence of involvement of adjacent structures. **(C)** Axial pelvic MRI showing a right adnexal mass measuring approximately 65 mm × 55 mm × 62 mm, with relatively homogeneous internal signal and no associated pelvic lymphadenopathy or ascites.

The primary indication for surgery was the inability to exclude malignancy in a progressively enlarging solid adnexal mass. The patient's perimenopausal status, persistent abnormal vaginal bleeding, and lack of fertility requirements were additional considerations. Laparoscopic total hysterectomy and bilateral salpingo-oophorectomy were therefore performed on January 3, 2025. Intraoperative findings (Figure 2) revealed a normal-sized uterus, a solid right ovarian mass with an intact capsule, an elongated right fallopian tube, and an unremarkable left adnexa. No pelvic adhesions or invasion of surrounding organs were observed. Frozen section examination of the right adnexal lesion demonstrated abundant endometrioid-like glandular structures with focal epithelial proliferation, suggesting a benign or borderline lesion of possible endometrial origin. Gross examination of the hysterectomy specimen is shown in Figure 3A. Histopathological examination showed proliferative endometrium and endometrial polyps. Chronic cervicitis with Nabothian cyst formation was also noted, and no adenomyosis was reported. Permanent paraffin-embedded histopathological examination of the right ovarian lesion revealed benign ovarian endometrioid adenofibroma characterized by endometrioid glands embedded within abundant fibrous stroma, with focal epithelial proliferation (Figure 3B). Immunohistochemical staining showed that the epithelial component was positive for PAX8, ER, and PR, with absence of PAX2 and PTEN expression; CK7 and CD10 were negative. The stromal component showed focal positivity for *α*-inhibin and diffuse positivity for SMA. The Ki-67 labeling index was approximately 10% (Figure 3C). Despite the focal epithelial proliferation, the absence of significant cytological atypia, increased mitotic activity, and destructive stromal invasion supported the diagnosis of benign ovarian endometrioid adenofibroma.

**Figure 2:**
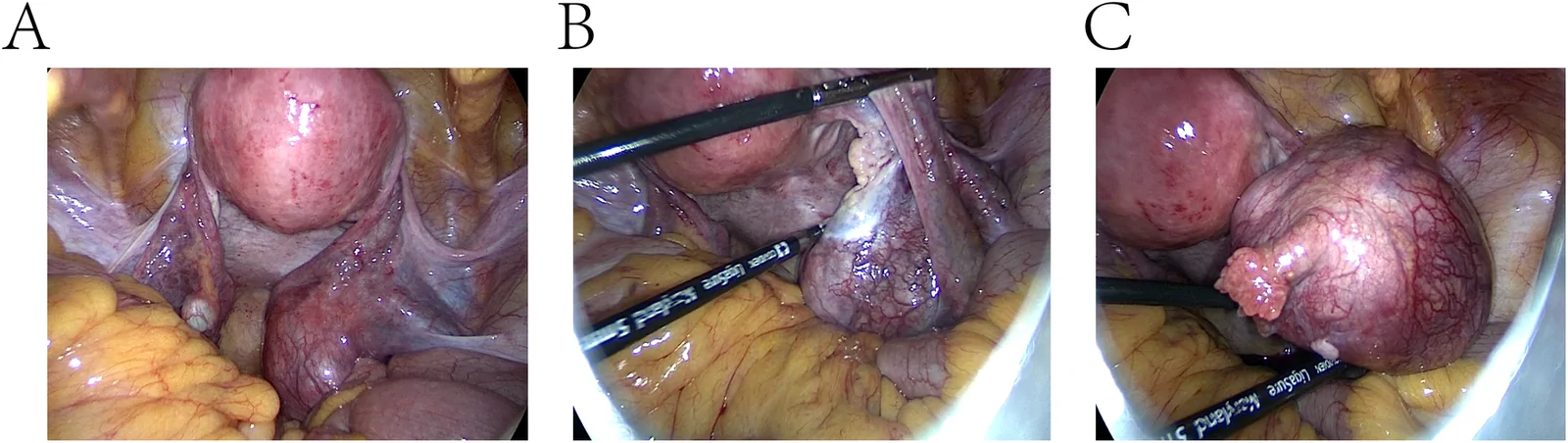
Intraoperative laparoscopic findings. **(A)** Normal uterine morphology with a smooth surface; a solid right ovarian mass with an intact capsule was identified, while the left adnexa appeared unremarkable. **(B)** The right ovarian mass was dissected and found to be intact, without adhesions to surrounding structures; the right fallopian tube appeared elongated. **(C)** Exposed right ovarian mass showing a solid and vascular appearance, with a clear boundary from the surrounding ovarian tissue and no gross evidence of invasion.

**Figure 3:**
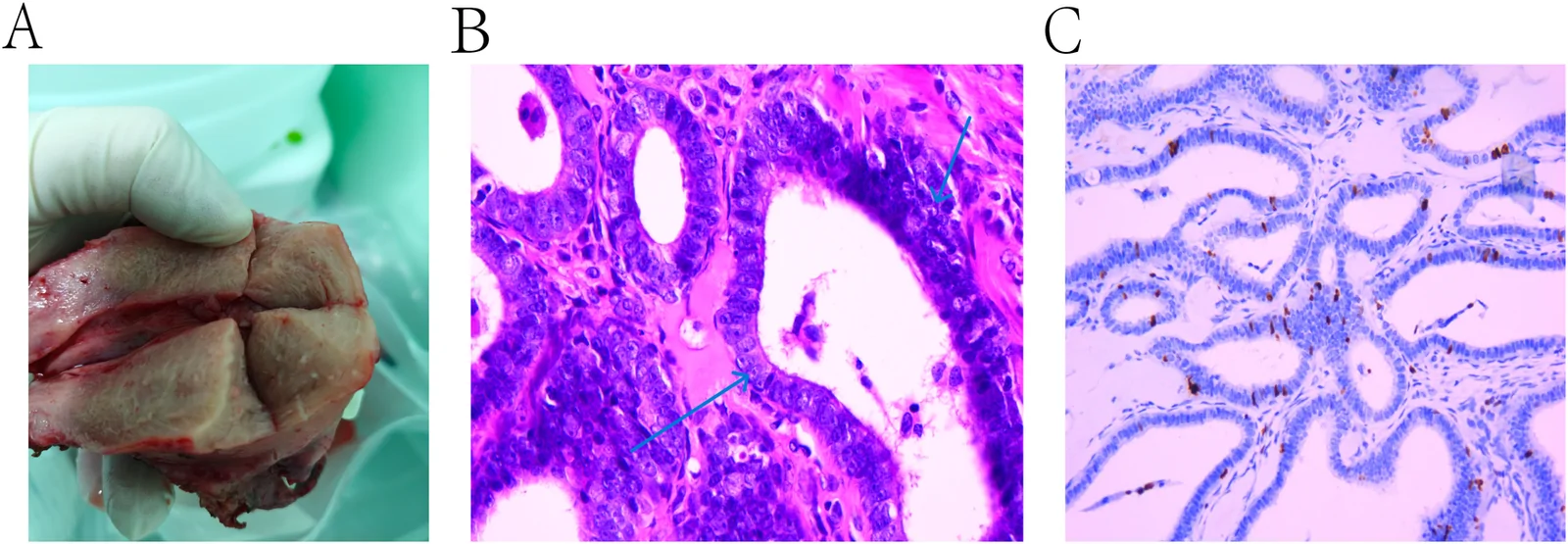
Gross uterine specimen and histopathological and immunohistochemical findings of the ovarian lesion. **(A)** Gross examination of the hysterectomy specimen showing the cut surface of the uterus. **(B)** Hematoxylin and eosin staining demonstrating endometrioid glands embedded within abundant fibrous stroma. Focal epithelial proliferation with increased glandular cellularity is indicated by arrows. No significant cytological atypia, increased mitotic activity, or destructive stromal invasion was identified. **(C)** Immunohistochemical staining for Ki-67 showing focal proliferative activity within the epithelial component (original magnification ×20). The Ki-67 labeling index was approximately 10%.

The patient recovered uneventfully after surgery and was discharged on January 6, 2025. During the 11-month follow-up period, the patient remained asymptomatic, and follow-up ultrasonography showed no evidence of recurrence or other abnormalities. The timeline of diagnosis and treatment is presented in Figure 4.

**Figure 4:**
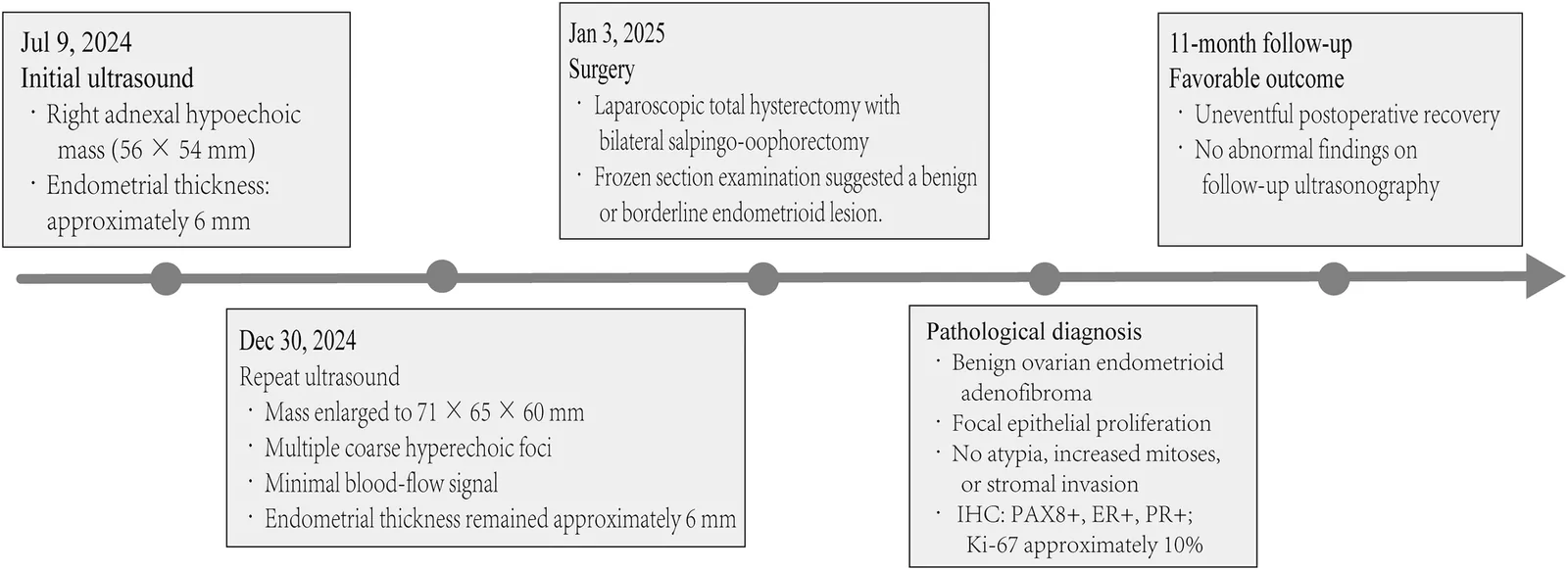
Timeline of diagnosis, treatment, and follow-up of the patient with ovarian endometrioid adenofibroma.

## Discussion

The concept of “adenofibroma” was first proposed by Ober in 1959 and refers to a group of biphasic epithelial–stromal neoplasms with Müllerian differentiation (6). Ovarian endometrioid adenofibroma (EA) is an exceptionally rare benign epithelial tumor of the ovary. According to the 2020 World Health Organization (WHO) Classification of Tumors of the Female Genital Organs, EA is classified as a benign tumor with endometrioid differentiation together with endometrioid cystadenoma (7). Its characteristic pathological feature is the coexistence of prominent fibrous stromal components and endometrioid glandular structures. Regarding tumor origin, EA is considered to arise from ovarian surface epithelium or cortical stromal tissue, although the exact origin remains uncertain. Müllerian/endometrioid epithelial differentiation may contribute to the development of this tumor phenotype. Meanwhile, some cases have been reported in association with ovarian endometriosis, suggesting that endometriosis may represent an associated condition or a potential contributor to tumor development in a subset of cases (1, 8, 9).

Despite this possible relationship, ovarian EA should be distinguished from endometriosis-associated lesions. Histologically, endometriosis is characterized by ectopic endometrial glands and stroma outside the uterine cavity, often accompanied by hemorrhage, hemosiderin-laden macrophages, and chronic inflammatory changes (10, 11). In contrast, EA demonstrates a characteristic adenofibromatous architecture, consisting of endometrioid glands of variable sizes lined by cuboidal or low columnar epithelium and embedded within abundant fibrous stroma (1, 2). The stromal component of EA is typically dense collagenized or hypercellular and lacks the hemosiderin deposition and endometrial-type stromal features commonly observed in endometriosis (1). Although mild epithelial crowding may occasionally be observed, benign EA lacks cytological atypia and destructive stromal invasion. Therefore, the diagnosis of EA relies primarily on recognition of its characteristic epithelial–stromal architecture and careful exclusion of endometriosis-associated lesions and other malignant epithelial ovarian tumors. Similarities between fimbrial adenofibroma and ovarian EA further suggest the diversity of Müllerian differentiation within tubo-ovarian tissues.

The clinical manifestations of ovarian EA are usually nonspecific. Most patients are asymptomatic or present with nonspecific symptoms such as lower abdominal discomfort or pelvic distension. Abnormal vaginal bleeding has occasionally been reported in ovarian endometrioid adenofibromatous or cystadenofibromatous tumors, particularly in hormonally functional tumors associated with estrogen excess and endometrial changes (12). In the present case, however, there was no evidence of estrogen excess, and the endometrial thickness remained approximately 6 mm on serial ultrasonography, providing no clear evidence that the ovarian tumor was hormonally functional. Therefore, the patient's intermittent vaginal bleeding could not be directly attributed to the ovarian EA. Postoperative histopathological examination of the uterus demonstrated proliferative endometrium and endometrial polyps, providing a plausible uterine explanation for the abnormal bleeding. Given the patient's perimenopausal status, anovulatory bleeding may also have contributed. Preoperative endometrial sampling was not performed, which represents a limitation of the diagnostic work-up and prevented definitive preoperative determination of the source of bleeding.

Regarding imaging findings, ultrasonography commonly demonstrates a cystic-solid adnexal mass, with solid components reflecting abundant fibrous stroma. MRI may provide additional diagnostic information, as the fibrous component may appear as relatively low signal intensity on T2-weighted images. However, imaging findings are not specific, and EA may mimic other benign or malignant adnexal tumors. Therefore, definitive diagnosis relies on histopathological examination supported by immunohistochemical evaluation. Previous studies have reported that epithelial components of EA may express CD10, whereas stromal CD10 expression is usually absent; stromal smooth muscle actin (SMA) expression may be focal, while calretinin and desmin are generally negative, which may assist in differentiating EA from endometriosis and adenomatoid tumors (13). In the present case, immunohistochemical staining showed diffuse positivity for PAX8, ER, and PR in the epithelial component, focal stromal *α*-inhibin expression, and diffuse stromal SMA positivity. The epithelial component showed absence of PAX2 and PTEN expression, while CK7 and CD10 were negative. The Ki-67 proliferation index was approximately 10%. Loss of PAX2 and PTEN expression may also occur in atypical endometrial hyperplasia/endometrioid intraepithelial neoplasia (AEH/EIN); therefore, these immunohistochemical findings are not diagnostically specific when interpreted in isolation (14, 15). Accordingly, the distinction in the present case relied primarily on the anatomical location and characteristic histomorphology. The ovarian lesion demonstrated an adenofibromatous architecture consisting of endometrioid-type glands embedded within abundant fibrous stroma, without significant cytological atypia, increased mitotic activity, or destructive stromal invasion. Taken together, these morphological findings supported the diagnosis of benign ovarian endometrioid adenofibroma, while the immunohistochemical findings were regarded as ancillary rather than independently diagnostic.

In the present case, surgical management was determined based on the patient's age, menopausal transition status, presence of a solid adnexal mass, and the inability to exclude malignancy preoperatively. For peri- or postmenopausal patients without fertility requirements, definitive surgery may be considered when malignancy cannot be ruled out. However, fertility-sparing approaches may be considered in younger patients with benign-appearing lesions and a desire for future fertility. Therefore, treatment decisions should be individualized according to patient characteristics, clinical presentation, and the level of suspicion for malignancy.

The prognosis of benign EA is generally favorable, with recurrence being uncommon after complete excision. However, rare atypical, proliferative, borderline, or malignant counterparts have been described (16, 17). Careful histopathological evaluation is therefore essential, particularly in tumors showing increased glandular complexity, significant cytological atypia, destructive stromal invasion, or elevated proliferative activity (2, 7). Rare associations and potential biological relationships between EA and endometrioid carcinoma have been reported. In a previous study of ovarian endometrioid adenocarcinomas with infiltrative “adenofibroma-like” morphology, benign endometrioid adenofibroma components were identified in some cases, suggesting possible overlapping biological features among these lesions rather than a predictable progression pathway (18). Similarly, rare associations between EA and mesonephric-like adenocarcinoma have been reported; however, these observations remain limited to isolated cases and do not establish a direct causal relationship (4, 5).

## Conclusion

Ovarian endometrioid adenofibroma is an exceptionally rare benign ovarian tumor with nonspecific clinical manifestations. Imaging findings may suggest an adnexal mass but are insufficient for definitive diagnosis, which mainly relies on careful histopathological evaluation and immunohistochemical examination. Recognition of the characteristic epithelial–stromal architecture is essential for distinguishing EA from endometriosis-associated lesions and other malignant ovarian tumors. The prognosis of benign EA is generally favorable, and management should be individualized according to patient age, fertility considerations, clinical presentation, and the possibility of malignancy. Clinicians should remain aware of rare atypical, borderline, or malignant counterparts and possible associations with endometriosis or endometrial lesions.

## Patient perspective

The patient reported that intermittent vaginal bleeding for nearly one year was the main reason she sought medical attention. She described feeling worried about the cause of the bleeding and the possibility of a serious disease. After discussion of the imaging findings, surgical plan, and expected outcomes, she felt reassured and agreed with the treatment. Following surgery, she recovered well and was relieved by the benign pathological diagnosis. At follow-up, she reported no new symptoms and had returned to her usual daily activities.

## Data Availability

The original contributions presented in the study are included in the article/Supplementary Material, further inquiries can be directed to the corresponding authors.
