# Supplementary material for: Ovarian endometrioid adenofibroma in a perimenopausal woman with intermittent vaginal bleeding: a case report
Source: Front Reprod Health. 2026 Sep 28;8:1839813. doi: 10.3389/frph.2026.1839813 (PMC13655068; doi:10.3389/frph.2026.1839813)
Supplement: Supplementary file 1 [file Datasheet1.pdf]

Supplementary Figure S1

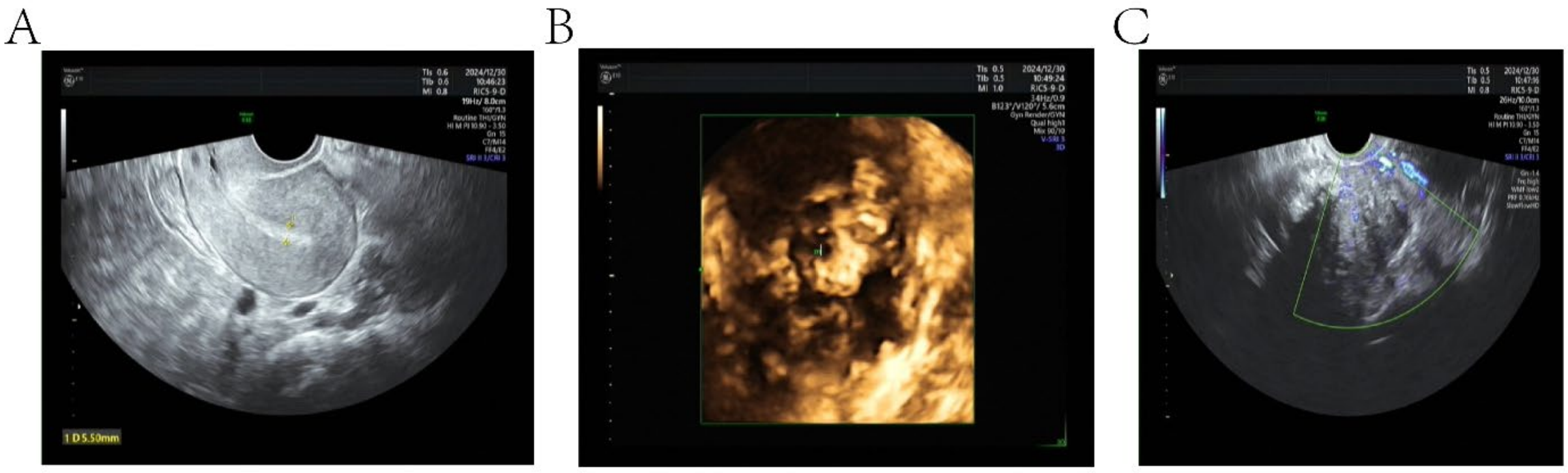

Preoperative transvaginal ultrasonography obtained on December 30, 2024. (A) Grayscale ultrasonography showing a hypoechoic mass in the right adnexal region. (B) Three-dimensional ultrasonography demonstrating the internal appearance of the lesion. (C) Color Doppler imaging showing minimal internal blood flow. The lesion measured approximately 71 × 65 × 60 mm and was interpreted as a solid right adnexal mass with multiple coarse hyperechoic foci.
